# Multi-Objective Optimization of Mechanical and Geometric Properties of 3D-Printed PLA Porous Scaffolds for Biomedical Applications

**DOI:** 10.3390/ma19051008

**Published:** 2026-03-05

**Authors:** Alejandro González González, Patricia C. Zambrano-Robledo, Deivis Avila, Marcelino Rivas, Ramón Quiza

**Affiliations:** 1 Centro de Estudio de Fabricación Avanzada y Sostenible (CEFAS), Universidad de Matanzas, Matanzas 40100, Cuba; alejandro.glez@umcc.cu (A.G.G.); marcelino.rivas@umcc.cu (M.R.); ramon.quiza@umcc.cu (R.Q.); 2 Centro de Innovación e Investigación en Ingeniería Aeronáutica, Facultad de Ingeniería Mecánica y Eléctrica, Universidad Autónoma de Nuevo León, San Nicolás de los Garza 66455, NL, Mexico; patricia.zambranor@uanl.edu.mx; 3 Escuela Politécnica Superior de Ingeniería, Universidad de La Laguna (ULL), Avenida Ángel Guimerá Jorge, s/n. 456-38200, 38200 Santa Cruz de Tenerife, Tenerife, Spain

**Keywords:** fused deposition modeling, triply periodic minimal surfaces, biomedical scaffolds, response surface methodology, multi-objective optimization, objective reduction

## Abstract

Porous scaffolds fabricated via fused deposition modeling (FDM) are promising for bone tissue engineering, but their mechanical performance and geometric fidelity are governed by complex interactions between process parameters and architectural design. This study presents a multi-objective optimization framework for poly (lactic acid) (PLA) scaffolds based on three triply periodic minimal surface (TPMS) topologies—Gyroid, Primitive, and Diamond. A Box–Behnken design combined with response surface methodology was used to model compressive strength, elastic modulus, yield strength, energy absorption density, and discrepancies in volume and porosity as functions of layer thickness (0.05–0.15 mm), extrusion temperature (210–220 °C), and target porosity (50–70%). The resulting quadratic models exhibited strong predictive capability (R^2^ > 77%, with most >90%) and were validated experimentally at extreme parameter combinations, yielding relative errors below 10% for 83% of measurements. Multi-objective optimization using NSGA-II, coupled with principal component analysis and correlation-based objective reduction, revealed that the six original objectives collapse to topology-specific essential pairs: absorbed energy density and porosity discrepancy for Gyroid; Young’s modulus and volume discrepancy for Primitive; and Young’s modulus and porosity discrepancy for Diamond. The generated Pareto fronts quantify the inherent trade-off between mechanical performance and geometric fidelity for each topology, providing designers with explicit decision maps. This framework enables rational, application-driven selection of printing parameters and scaffold architecture, advancing the clinical translation of patient-specific FDM-printed bone scaffolds.

## 1. Introduction

Porous scaffolds are three-dimensional structures fabricated from biomaterials, serving as support templates for tissue grafts in the human body. To be suitable for biomedical applications, they must exhibit specific surface, biological, geometric, and mechanical properties [1]. Key requirements include high surface area and surface-to-volume ratio, alongside a chemical composition, pH, and surface charge that enable cell adhesion and proliferation [2]. Furthermore, scaffolds must be biocompatible and biodegradable, with a degradation rate matching the speed of new tissue formation [3]. From a geometric perspective, scaffolds require adequate porosity and permeability, with a highly interconnected pore network to facilitate the transport of nutrients and cellular waste removal [4,5]. Ideal scaffold porosity typically ranges from 60% to 90%, though the optimal pore size varies significantly depending on the target tissue [6]. For instance, muscle tissue growth is favored by pore sizes of 50–200 µm, while bone tissue requires 200–400 µm [3], with a general minimum of 100 µm needed for vascularization [7], though these values are not absolute [8,9]. Mechanically, scaffolds must possess properties that allow them to maintain the structure and function of the native tissue they aim to replace [10]. This necessitates more elastic and less rigid structures for tissues like skin or cartilage [11], and higher stiffness and strength for load-bearing applications such as bone implants [12,13,14].

The scaffold concept was introduced in the mid-1980s and has since become a cornerstone of tissue engineering [15]. Today, scaffolds find diverse biomedical applications, including skin regeneration [11], breast tissue engineering [16], dental applications [7,17], cartilage and maxillofacial surgery [10], cancer treatments [17,18], and bone regeneration—one of the most studied areas [12,13,19,20,21,22,23]. The integration of gene-editing technologies, stem cells, nanotechnology, and bioprinting enables the creation of sophisticated devices that closely mimic living tissue [24]. A primary limitation, however, remains the precise adaptation to the native tissue, particularly in matching mechanical properties to avoid stress shielding and ensuring sufficient vascularization [14,25].

The construction of porous scaffolds relies on three factors: material selection, internal structure design, and the manufacturing method. Among the various materials, polymers hold a prominent position. Poly (lactic acid) (PLA), an aliphatic polyester, is one of the most widely used thermoplastic polymers in biomedical applications [8,26,27,28,29,30]. Its popularity stems from its good processability, biodegradability, biocompatibility, relatively low production cost, and lower Young’s modulus compared to metals, which helps reduce stress shielding [27,31]. It is also approved by the FDA for use in biomedical implants [32]. Its main disadvantages are low toughness, slow degradation rate, and relatively high hydrophobicity, which can be mitigated via coatings, composites with bioceramics like hydroxyapatite, or copolymerization [33,34].

Manufacturing methods are divided into conventional and additive manufacturing (AM) techniques. Conventional methods like solvent casting/particulate leaching and electrospinning often suffer from poor control over pore architecture, the use of toxic solvents, and low reproducibility [1,35]. In contrast, AM, or 3D printing, builds scaffolds layer-by-layer from a digital model, offering superior control over geometry (porosity, pore size, interconnectivity), ease of operation, and the ability to create patient-specific constructs with complex geometries [35,36]. Fused Deposition Modeling (FDM) is a prominent, cost-effective AM technique with high print speeds and easy maintenance [36,37]. In FDM, a thermoplastic filament is heated, extruded through a nozzle, and deposited onto a platform. The process does not require organic solvents or post-curing, making it suitable for various polymers and composites [11].

Computational scaffold design overcomes the limitations of trial-and-error experimental approaches, enabling control over geometry and properties and allowing simulations (e.g., Finite Element Analysis) before fabrication [38,39,40]. Designs can be classified as non-parametric (e.g., based on CAD, stochastic, or image-based), which are often time-consuming and offer limited control [41,42,43], and parametric designs, generated by computational algorithms, which provide greater design flexibility.

Among parametric designs, Triply Periodic Minimal Surfaces (TPMS) have gained significant interest [44,45]. A minimal surface has zero mean curvature, a property found in some natural tissues like bone. TPMS structures, such as the Primitive (P), Gyroid (G), and Diamond (D) surfaces, exhibit high surface-to-volume ratios, full interconnectivity, and superior permeability compared to non-parametric designs [46,47]. Their mathematical description via simple trigonometric equations facilitates computational modeling [48], and they have been extensively used as the basis for scaffolds in various biomedical studies [5,49,50,51,52,53,54].

The combination of PLA as the material, TPMS as the parametric design method, and FDM as the manufacturing technique constitutes a viable and powerful strategy for creating customized biomedical scaffolds with controlled internal architectures and tailored properties.

The quality of FDM-printed objects, including scaffolds, is critically influenced by printing parameters, which affect both mechanical performance and dimensional accuracy (the discrepancy between the printed object and its digital design) [55,56]. Key mechanical properties studied include tensile, compressive, and flexural strength, while dimensional accuracy encompasses deviations in length, width, and surface roughness. The most influential FDM process parameters are layer thickness, printing orientation, raster orientation, infill density, print speed, and extrusion temperature [43,55,57,58,59,60,61].

To model the relationship between these parameters (independent variables) and the resulting properties (responses), researchers employ experimental designs coupled with statistical analysis. The most common approaches are the Taguchi method, full and fractional factorial designs, Central Composite Designs (CCD), and Box–Behnken Designs (BBD), often paired with Analysis of Variance (ANOVA), Response Surface Methodology (RSM), or Artificial Neural Networks (ANN) [55]. For instance, Taguchi with ANOVA has been used to optimize tensile strength and dimensional accuracy [62,63,64], and also to model hardness and tensile strength [65] and to analyze elastic modulus, ultimate strength, and elongation [66].

BBD with RSM has been applied to model surface roughness and compressive strength as functions of platform temperature, printing speed, and layer thickness [67], as well as to model density, manufacturing time, surface roughness, and micro-hardness as functions of infill density, layer thickness, and platform temperature [68]. Another approach applied BBD coupled with ANFIS and ANN models to investigate the influence of raster orientation, printing orientation, air gap, raster width, and layer thickness on the tensile and flexural strength of ABS specimens [69], while further work modeled the influence of infill density, extrusion temperature, and printing speed on the tensile strength of PLA specimens [70]. Comparative studies indicate that BBD achieves mean square error and absolute error values very close to those of full factorial designs, using only 15 experiments versus 81, making it an efficient choice [71].

A significant limitation in the literature is that most modeling and optimization studies focus on solid test specimens (e.g., “dog-bone” shapes) rather than porous scaffolds. Factors such as infill density and pattern—relevant for solid objects—are not applicable to scaffolds, where porosity is defined by the CAD design. Furthermore, there is a scarcity of studies that model scaffold properties while simultaneously considering the coupled influence of both printing parameters (e.g., layer thickness, extrusion temperature) and design parameters (TPMS type and target porosity). This constitutes a critical research gap.

Equally important is how the resulting models are used for optimization. Studies that do address scaffold properties often rely on scalarization techniques—such as Gray Relational Analysis, desirability functions, or signal-to-noise ratios—which combine multiple objectives into a single function and yield a single optimal solution rather than a true Pareto-optimal set [65,72,73,74,75,76]. While computationally simple, these methods fail to capture trade-offs between conflicting objectives like mechanical strength and geometric fidelity. Multi-objective optimization (MOO) approaches, based on evolutionary algorithms such as the Non-dominated Sorting Genetic Algorithm II (NSGA-II), are better suited for such problems, as they generate a set of Pareto-optimal solutions that explicitly represent design trade-offs [77,78,79,80,81,82,83]. Within this framework, objective reduction techniques based on principal component analysis and statistical correlations can further enhance performance by identifying and removing redundant objectives, thereby reducing computational cost and improving solution interpretability [84,85,86]. Quality metrics like the hyper-volume indicator provide robust means to evaluate the convergence and diversity of the resulting Pareto fronts [87,88].

Therefore, a research niche exists for implementing a comprehensive a posteriori multi-objective optimization strategy—incorporating objective reduction and Pareto front analysis—that simultaneously models and optimizes multiple mechanical and geometric properties of porous PLA scaffolds, considering the coupled influence of TPMS architecture (Gyroid, Primitive, Diamond), design porosity, and key FDM process parameters (layer thickness and extrusion temperature). The hyper-volume metric can serve as a robust performance indicator for such an approach.

Consequently, the objective of this research was to develop and validate a comprehensive methodological framework that addresses this gap by combining design of experiments, response surface modeling, and multi-objective optimization with objective reduction to enable the identification of optimal parameter combinations for TPMS-based scaffolds manufactured via FDM.

The article is structured as follows. Following this introduction, Section 2 describes the materials and methods, including computational scaffold design, FDM manufacturing, design of experiments, response variables, experimental tests, and the multi-objective optimization problem formulation. Section 3 presents and analyzes the results, covering regression model development and validation, physical interpretation of mechanical and geometric behavior, multi-objective optimization outcomes, objective reduction, and Pareto front analysis for each TPMS topology. Section 4 presents the conclusions and discusses the implications of the results, together with future research directions.

## 2. Materials and Methods

The workflow for this research is as follows:

1. Computational design of the TPMS structures using the parametric method to generate STL files for each structure with different porosities.

2. FDM printing, with layer thickness and extrusion temperature varied according to a DOE. Three replicates were obtained for each combination of these three factors (porosity, temperature, and layer thickness) and used to determine their dimensions and relate them to volume and porosity discrepancies, as well as for compression experiments.

3. Modeling of each response using response surface methodology, followed by physical, statistical and experimental validation.

4. Multi-objective optimization and determination of essential versus redundant/correlated objectives.

### 2.1. Scaffold Design and Fabrication

The mathematical equations for modeling TPMS can be obtained from nodal approximations to Fourier series of minimal surfaces of interest (Gandy2001). In this work, we use the Gyroid (G), Primitive (P), and Diamond (D) types of TPMS:

(1) $$P:\cos\left(X\right)+\cos\left(Y\right)+\cos\left(Z\right)+C_{P}=0$$

(2) $$G:\cos\left(X\right)\sin\left(Y\right)+\cos\left(Y\right)\sin\left(Z\right)+\cos\left(Z\right)\sin\left(X\right)+C_{G}=0$$

(3) $$D:\cos\left(X\right)\cos\left(Y\right)\cos\left(Z\right)-\sin\left(X\right)\sin\left(Y\right)\sin\left(Z\right)+C_{D}=0$$

where $\left(X,Y,Z\right)=\left(n_{x}\pi x,n_{y}\pi y,n_{z}\pi z\right)$ and each $n_{i}$ allows us to control periodicity, pore size and unit cell size in every spatial direction. Specimens were designed with dimensions 12.7 × 12.7 × 25.4 mm, according to ASTM D695-15 [89], comprising 6 × 6 × 12 unit cells with an approximate linear size of 2.12 mm. Parameters (CP, CG, CD) define the C-level set for porosities of 50, 60, and 70% according to Table 1.

All 3D computational models were created using Wolfram Mathematica v11.3, and the STL files were repaired and smoothed using AutoDesk Meshmixer v3.4.474 (AutoDesk, Inc. 2017, San Rafael, CA, USA).

The material used in the manufacture of the scaffolds is natural white commercial PLA in filament form with a diameter of 1.75 ± 0.03 mm from Smart Materials 3D (Jaén, Spain). The manufacturer reported that this brand of PLA has a density of 1.24 g/cm^3^, a melting point of 175 °C, and a glass transition temperature in the range of 55–60 °C.

Scaffold specimens were fabricated by FDM using a Wanhao Duplicator 6 (Wanhao 3D Printer, Jinhua, China) printer running Ultimaker Cura v4.8.0 software for slicing and parameter setting. The printing orientation was fixed, with the longest specimen dimension aligned with the vertical Z axis. Default printing parameters are shown in Table 2.

### 2.2. Design of Experiments

The design of experiments comprises the selection of factors, response variables, and experimental design type.

#### 2.2.1. Factors for Experimental Design

We conducted an analysis of the influence of printing parameters on the mechanical properties and dimensional accuracy of objects fabricated using the FDM process, based on seven review articles.

**Figure 1:**
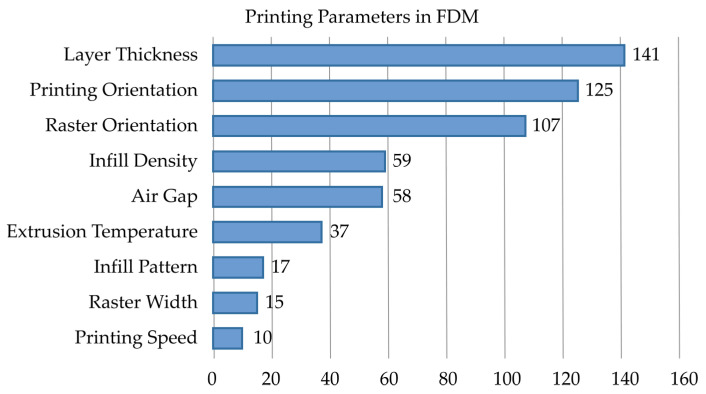
Relative importance of FDM printing parameters based on literature frequency.

In Table 3, “Total” refers to the total number of papers consulted in every review, while “Useful” refers to how many of those were useful to our research. Figure 1 relates the total number of papers that mention or use the corresponding factor, where the two most influential factors are layer thickness and printing orientation. However, in this work we fixed the orientation, while raster orientation has no useful meaning because we are printing a TPMS structure. The infill density is always 100% and the air gap is always zero. As a result, in this case, the two experimental factors (printing parameters) are the layer thickness and the printing temperature.

On the other hand, geometrical factors also affect the mechanical properties of scaffolds. In this work, we study the influence of porosity and type of TPMS (Gyroid, Diamond, Primitive) on the mechanical and geometrical properties of scaffolds. Therefore, we have a total of four variables for our experimental design: porosity, TPMS type, layer thickness, and printing temperature.

#### 2.2.2. Response Variables

**Figure 2:**
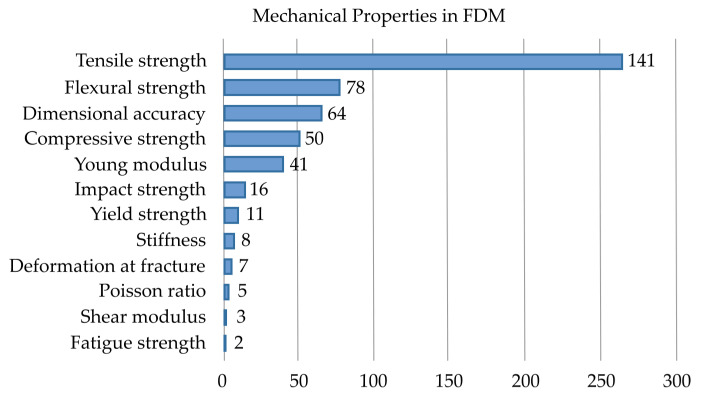
Order of importance of mechanical and geometrical properties in FDM.

Table 3 also informed the selection of response variables. Figure 2 represents the most studied mechanical properties along with the overall studies on dimensional accuracy.

According to the results shown in Figure 2, we selected six response variables: compressive strength ($\sigma_{C}$), yield strength ($\sigma_{Y}$), elastic modulus (E), density of energy absorption (U), volume discrepancy (ΔVol), and porosity discrepancy (ΔPor). The density of absorbed energy was included due to its importance to TPMS structures [53,90,91]. The fifth and sixth responses quantify dimensional accuracy as the relative discrepancy between measured (experimental) and designed (theoretical) values.

#### 2.2.3. Experimental Design

Based on the characteristics of this research, a central composite design (CCD) would not be suitable because the star points fall outside the safe operating range of the parameters and have values that are not experimentally achievable. The Box–Behnken design allows for the efficient estimation of linear and quadratic coefficients, as well as pure error and lack of fit, using fewer runs than the CCD, which translates into less printing material and less experimentation time.

From a mathematical perspective, response surface models are a less complex alternative to neural networks or group method of data handling (GMDH). Furthermore, they allow for modeling main effects and interactions up to the second order with fewer experiments, thus saving time and experimental resources compared to full factorial designs. The ability to increase the sensitivity of predictions by adding central points to determine curvature is another strength of response surface designs. In particular, the Box–Behnken design allows fitting quadratic surfaces with up to 21 factors without loss of efficiency and is the appropriate design when points at the edges of the experimental space are not required [92].

We chose a Box–Behnken design [93,94] coupled with Response Surface Methodology (RSM), as shown in Table 4. In the case of TPMS type, it is a nominal (categorical) variable modeled using two binary variables: P and G, with P = 1, G = 0 for Primitive, P = 0, G = 1 for Gyroid, and P = 0, G = 0 for Diamond.

The RSM is designed to fit a second-order polynomial. To decrease multicollinearity [94], factors were coded in [−1, 1] using the following:

(4) $$\hat{x}=\frac{2\;x-\left(x_{H}+x_{L}\right)}{x_{H}-x_{L}}$$

where x is the variable with physical units, $\hat{x}$ is the coded non-dimensional variable, and ($x_{H,}x_{L}$) are the high (*H*) and low (*L*) values of the factor. For each combination of structure type and numerical factors, we obtained 45 experiments (15 for each TPMS with three replicates), giving a total of 135 experiments. All regression models and statistical significance tests were conducted in Minitab v20.3 with a confidence level of 95%.

The extrusion temperature range was selected based on the manufacturer’s recommended printing temperature for PLA (205–220 °C). Moreover, preliminary printing experiments indicated that printing at 230 °C and above caused severe scaffold distortion, and the internal TPMS structures were completely lost. The range for layer thickness is determined through trial and error in preliminary printing experiments to verify, via visual inspection, that there is no severe warping of the structures to be printed or clogging of the print nozzle.

#### 2.2.4. Experimental Validation

The experimental validation of the regression models was carried out at the extreme points of the factor space. These points were chosen because the BBD does not consider them during the model building phase, and these models should be able to predict responses even at these extreme points of the design space, which are part of the independent variable ranges.

These points are located at the corners of the parameter space. For each structure type (D, P, and G), 8 points (the vertices of the cube) were taken, resulting in a total of 24 validation points. For each experimental combination defining these points, the corresponding experiments were performed, and the properties were calculated. The relative percentage error was computed as the difference between the measured property and the model-predicted value, divided by the measured value.

### 2.3. Experimental Work

Compression tests were conducted using a universal test machine Shimadzu AG-X Plus (Shimadzu Corp., Kyoto, Japan) with a 100 kN load cell, a loading speed of 0.5 mm/min, and a sampling frequency of 100 Hz. Experiments were randomized and the data were toe corrected. The energy absorption density was determined from the area under the stress–strain curve by numerical interpolation over the data points up to a strain of 0.4 [95].

Length, height, and depth dimensions of specimens were measured using a Mitutoyo digital caliper (uncertainty: 0.01 mm) and combined to calculate the experimental volume (V_Exp_) using the prism formula. The theoretical volume (V_Teo_) was calculated from the nominal dimensions specified by the ASTM standard. Porosity was determined using the Archimedean method to obtain the experimental porosity (ϕ_Exp_). For this, we used a mixture of deionized water (CIIIA Laboratories, Monterrey, Mexico) with 1% ethanol (CTR Scientific, Monterrey, NL, México), 99.6% pure. Samples were weighed in the air (m_A_) using an analytical balance OHAUS-Discovery of 0.001 g and then submerged in the mixture at 26 °C and weighed (m_B_). So, the experimental porosity is given by the following:

(5) $$\phi_{Exp}=\left(1-\frac{V_{Sol}}{V_{Teo}}\right)\times 100\%$$

(6) $$V_{Sol}=\alpha \frac{\left(m_{A}-m_{B}\right)}{\rho_{Mix}^{26\;{}^{\circ}C}-\rho_{Air}^{26\;{}^{\circ}C}}$$

where α = 0.9882 is the volume correction factor, $\rho_{Air}^{26\;{}^{\circ}C}=1.2\;kg/m^{3}$, and the density of the mixture is as follows:

(7) $$\rho_{Mix}^{26\;{}^{\circ}C}=\frac{\rho_{H_{2}O}^{26\;{}^{\circ}C}+0.01\rho_{ethanol}^{26\;{}^{\circ}C}}{1.01}$$

where $\rho_{H_{2}O}^{26\;{}^{\circ}C}=996.86\;kg/m^{3}$ and $\rho_{ethanol}^{26\;{}^{\circ}C}=784.1\;kg/m^{3}$ are the densities of water and ethanol, respectively, at 26 °C and normal atmospheric pressure [96].

Calculation of the discrepancies in porosity and volume follows the formulae:

(8) $$∆Vol=\left(\frac{V_{Exp}-V_{Teo}}{V_{Teo}}\right)\times 100\%$$

(9) $$∆\phi =\left(\frac{\phi_{Exp}-\phi_{Teo}}{\phi_{Teo}}\right)\times 100\%$$

### 2.4. Multi-Objective Optimization

In the context of scaffold fabrication for biomedical applications, the general goal is to obtain a mechanically optimal product that conforms to specifications [74,97]. So, we need to maximize the mechanical properties and minimize the porosity and volume discrepancies. The optimization problem is formulated as shown in Table 5. In this work, the decision variables are the experimental factors, and the objectives are the response variables. The optimization problem is defined as multi-objective with constraints on the decision variable ranges.

The nature of the formulated optimization problem can lead to conflicting objectives and determines the use of an evolutionary meta-heuristic approach [98]. This is a mixed-variable optimization problem [99]; we chose to reduce its computational complexity by solving for each TPMS structure separately and working only with continuous variables.

We used the MATLAB built-in NSGA-II algorithm coupled with an objective reduction scheme based on principal component analysis (PCA) and correlation [84,85] to optimize all six properties and classify them as essential or redundant.

The codes for multi-objective optimization and the implementation of the objective reduction scheme were developed in MATLAB v2020. Table 6 summarizes the modified and default parameter values for the NSGA-II algorithm.

As a measure of the quality of the 2D Pareto fronts, we used the hyper-area metric [88,100]. Optimization results include correlation matrices for each TPMS geometry after an initial algorithm run, scatter plots of the Pareto fronts, and plots of hyper-area versus number of generations for 10 experimental runs to demonstrate algorithm convergence.

## 3. Results and Discussion

### 3.1. Regression Models

For each mechanical and geometrical property of the PLA scaffolds, we developed quadratic regression models retaining only statistically significant terms (*p* < 0.05). All equations are decoded after back-transformation using Equation (4):

Compressive strength:

(10) $$\begin{array}{cc} \sigma_{C}=-840.511 & +\;2.8552\;G+874.168\;H-450\;H^{2}+23.2476\;P+8.512\;H\cdot P-1.3832\;\phi \\ & \;-0.0555\;G\cdot \phi -0.3572\;P\cdot \phi +7.9076\;T-3.6\;H\cdot T+0.0055\;\phi \cdot T\\ & \;-0.01832\;T^{2} \end{array}$$

Young’s modulus:

(11) $$\begin{array}{cc} E=-158,797\;+ & 357.6\;G+4908\;H-37,800\;H^{2}+1166.6\;P-4.42\;\phi -7.46\;G\cdot \phi \\ & +\;44.2\;H\cdot \phi -18.28\;P\cdot \phi +1479.2\;T-3.44\;T^{2} \end{array}$$

Yield strength:

(12) $$\begin{array}{cc} \sigma_{Y}=-3536.46 & +\;5.404\;G+200.22\;H-1064.8\;H^{2}+43.312\;P+12.74\;H\cdot P-0.016\;\phi \\ & -\;0.0999\;G\cdot \phi -0.2584\;P\cdot \phi +32.8824\;T-0.1336\;P\cdot T-0.07644\;T^{2} \end{array}$$

Absorbed energy density:

(13) $$\begin{array}{cc} U=-1531.86\;+ & 3.42\;G+89.922\;H-450\;H^{2}+10.6251\;P+4.258\;H\cdot P-0.00496\;\phi \\ & -\;0.057\;G\cdot \phi -0.17087\;P\cdot \phi +14.2416\;T-0.03312\;T^{2} \end{array}$$

Volume discrepancy:

(14) $$\begin{array}{cc} ∆V=1197.39\;- & 22.6209\;G+22.78\;H-21.64\;G\cdot H-143.2\;H^{2}-25.3703\;P-13\;H\cdot P\\ & +\;0.7458\;\phi -0.0227\;G\cdot \phi +0.526\;H\cdot \phi +0.0213\;P\cdot \phi -0.00639\;\phi^{2}\\ & -\;11.0614\;T+0.1302\;G\cdot T+0.117\;P\cdot T+0.02504\;T^{2} \end{array}$$

Porosity discrepancy:

(15) $$\begin{array}{cc} ∆\phi =1365.12\;+ & 23.368\;G-169.8\;H-16.08\;G\cdot H+788.4\;H^{2}-47.597\;P-18.1\;H\cdot P\\ & +\;0.6039\;\phi -0.0373\;G\cdot \phi -0.0835\;P\cdot \phi -0.0051\;\phi^{2}-12.7632\;T\\ & -\;0.0882\;G\cdot T+0.2566\;P\cdot T+0.02968\;T^{2} \end{array}$$

For each of the above equations, the categorical variables P and G can be used to obtain the corresponding equations for every type of TPMS. For example, setting P = 1, G = 0 gives the equations for Primitive (P), while P = 0, G = 0, corresponds to Diamond (D).

#### 3.1.1. Statistical Validation of Regression Models

Table 7 summarizes the most relevant statistical results for the modeling of mechanical and geometrical properties of the scaffolds. We initially performed regression with all possible terms, then retained only statistically significant terms (*p* < 0.05) at a 95% confidence level. In the worst case, the correlation coefficient exceeds 77%, indicating a moderate goodness of fit for all models. There is no statistical difference between $R^{2}$ and $R_{adj}^{2}$, indicating that there is no overfitting.

For every model, the residuals follow a normal distribution and the plots of the residuals vs. adjusted values indicate constant variance in all models. According to the Durbin–Watson test results, there is no correlation in regression residuals for adjacent observations, except in the case of the response “Porosity discrepancy” where the test is inconclusive [101]. For this case, we can only conclude that there is no first order negative correlation because 4-D_W_ = 2.23763 > D_U_ = 1.95112. In all models, the variance inflation factor (VIF) for model coefficients lies in the interval [1;1.5], indicating no correlation between predictors, so they are independent of each other.

#### 3.1.2. Physical Validation of Regression Models

The mechanical property values obtained experimentally correspond to reported values for human trabecular bones. Young’s modulus has been reported in the range 50–500 MPa for common load-bearing regions (e.g., mandible) [102], while the yield strength lies in the range 0.1–10 MPa for vertebrae and mandibles [102,103]. Our results are 100–500 MPa for Young’s modulus and 5–11 MPa for yield strength, both within reported values. Additionally, compressive strength has been reported in the range 0.1–30 MPa [104], while our results range from 5–15 MPa.

Analysis of the stress–strain curves for the three scaffold structures (P, D, and G) reveals distinct deformation mechanisms. Structure P exhibits an initial elastic region until approximately 3% strain, after which its strength increases to a peak at 15–20% strain before undergoing structural collapse via shear fracture at 45° within the unit cells [52,105]. This progression indicates a deformation mechanism dominated by contraction and buckling, consistent with structure P possessing the highest Young’s modulus [105].

In contrast, structures D and G display mechanical behavior typical of bending-dominated cellular solids [106,107,108]. Following the elastic region, these structures generally enter a prolonged stress plateau before a rapid stress increase in the densification zone, resulting in full deformation without fracture [105]. At higher porosity levels, increased brittleness can lead to structural collapse immediately after the elastic region, bypassing the plateau [109]. The compression curves for all three structures align with literature reports, confirming that this characteristic behavior is independent of the base material.

**Figure 3:**
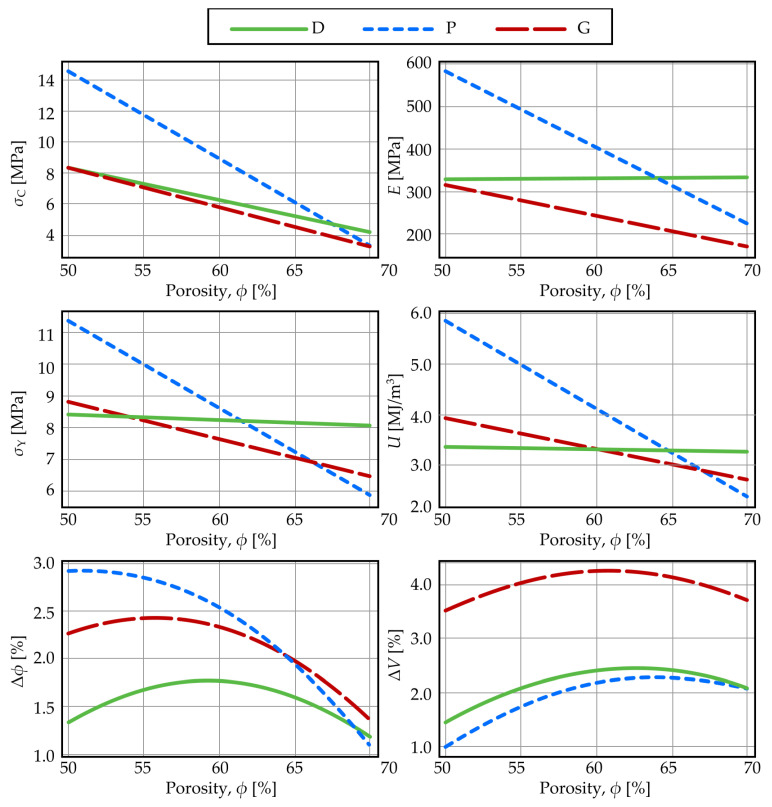
Mechanical and geometrical properties as functions of porosity.

Figure 3 illustrates the dependence of the mechanical and geometrical properties under study on porosity for the three structures, based on regression equations evaluated at a standardized layer thickness and extrusion temperature (H = 0.10 mm, T = 215 °C). As shown and corroborated by literature [106], structure P demonstrates superior elastic modulus and yield strength at low porosities. However, at porosities exceeding 60–65%, structure D begins to dominate, which is frequently reported as the strongest configuration [52,54]. As physically anticipated, all mechanical properties decrease with increasing porosity [105,108]. Structure D exhibits the smallest variation in its properties across the porosity range [110]. While structures D and G show similar porosity-dependent trends for strength and modulus, structure D consistently demonstrates higher values. This distinct behavior of structures D and G, compared to structure P, is consistent with their fundamental difference in deformation mechanism.

The energy absorption capacity of a cellular solid, such as a sheet-based triply periodic minimal surface (TPMS) structure, is governed by unit cell geometry (i.e., TPMS type), relative density (porosity), base material properties, and loading rate [95]. In this study, the use of a single material (PLA) and standardized quasi-static compression testing isolates the influence of porosity and structure type as the primary determinants of energy absorption, with all printing parameters held constant.

The deformation mechanism directly dictates energy absorption performance. P-type structures undergo shear fracture and collapse at 3–4% strain. In contrast, D- and G-type structures, which deform via a bending-dominated mechanism, exhibit an extended plateau region beyond the elastic limit. Since absorbed energy density is the integral of the stress–strain curve, the substantial area under this plateau for structures D and G typically exceeds the energy contributed by the sharp stress peak before fracture in structure P. Consequently, under full deformation, structures D and G demonstrate superior toughness, as established in prior work [53]. For consistency in comparative analysis, this investigation defines a fixed strain limit of 4% for calculating absorbed energy density. This criterion intentionally excludes the post-yield plateau contribution for D and G structures. Figure 3 illustrates the resulting trends: at low porosities (<65%), structure P exhibits the highest energy absorption, driven by its pronounced peak stress. However, for porosities above 65%, this relationship inverts. Structure D becomes predominant, followed by G and then P, a finding consistent with literature reports where analyses are typically conducted at high porosities (70–90%) and low strain limits (~6%) [53,111,112].

In this way, the regression models developed for each mechanical property yield curves that align with the physically expected porosity-dependent behavior. This coherence between the statistical models and the mechanistic analysis of stress–strain responses validates the reliability of the regression results.

Layer thickness is a printing parameter frequently studied in combination with other parameters [55], like the nozzle diameter, raster width, and the total number of layers in a piece [55,56]. It has variable effects on mechanical properties, mainly compressive strength and elastic modulus. It is generally accepted that an increase in layer thickness improves mechanical strengths [57], while a decrease improves dimensional accuracy by decreasing the calculated discrepancies of volume and porosity, reducing the typical staircase effect of specimens fabricated by FDM [58,113]. This occurs because, for a fixed part height, a decrease in layer thickness increases the total number of deposited layers, creating a temperature gradient that affects the lower layers. This improves interlayer diffusion and strengthens the bonding between layers, thereby enhancing mechanical strength. However, a larger number of layers results in more heating/cooling cycles, increasing thermal residual stresses, which cause distortions (warping) and interlayer fracture, thus reducing mechanical strength. This is reflected in the literature as a tendency to maintain intermediate values of the layer thickness [55].

**Figure 4:**
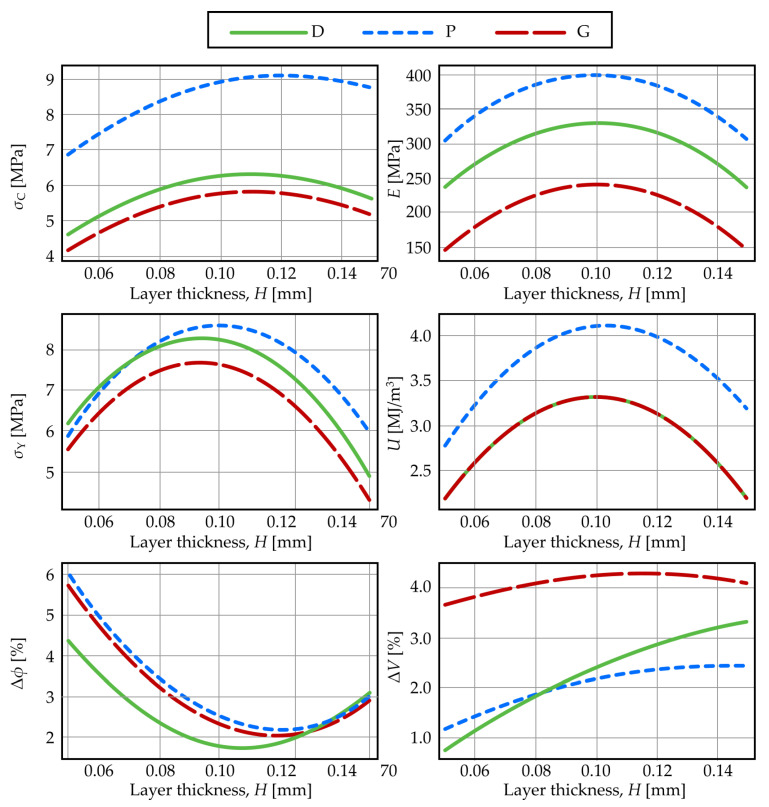
Mechanical and geometrical properties as functions of layer thickness.

Figure 4 shows the mechanical and geometrical properties (Equations (10)–(15)) as functions of layer thickness, with temperature and porosity held at their mid-range values (T = 215 °C, ϕ = 60%). The results indicate an optimal layer thickness for mechanical performance: properties are suboptimal at lower thicknesses, increase to a maximum, and then decrease at higher thicknesses. For porosity and volume discrepancies, the behavior is the opposite.

We hypothesize that the non-monotonic behavior of the porosity discrepancy, particularly the increase at the smallest layer thickness, is not solely a result of thermal residual stresses. Instead, it is likely dominated by a loss of geometric fidelity in the delicate lattice struts. At very low layer thickness, the thin extruded struts that form the lattice structure have insufficient self-support and are prone to sagging or displacement by the nozzle before solidification. This partial collapse of the intended porous architecture would increase the measured porosity error (Δφ), while at high layer thickness, the error is dominated by the coarse staircase effect. The minimum Δφ at intermediate layer thickness, therefore, represents the optimal balance between structural collapse at fine resolutions and geometric approximation errors at coarse resolutions.

The extrusion temperature can influence the fluidity and solidification characteristics of the molten material and control the viscosity of the extruded filament [55]. High extrusion temperatures ensure complete melting of all crystalline phases of the material and greater mobility of the polymer chains, which favors increased crystallinity due to a longer time for nucleation and crystal growth during the cooling process to room temperature [114,115]. Low extrusion temperatures can prevent the material from melting properly, causing nozzle clogging [55]. The literature reports that high extrusion temperatures improve mechanical properties, especially the strength, the elastic modulus, and the yield strength, while low extrusion temperatures improve dimensional accuracy. However, an excessively high extrusion temperature, close to the point at which thermal degradation of the material begins, can cause filament molding failures and dimensional inaccuracies [55].

**Figure 5:**
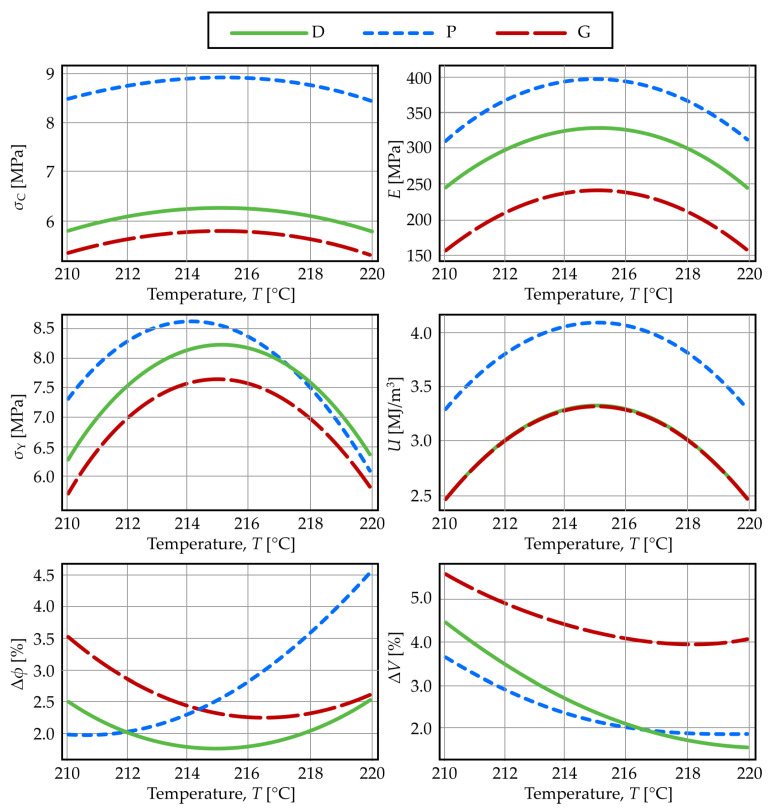
Mechanical and geometrical properties as functions of the extrusion temperature.

Figure 5 shows the mechanical and geometrical properties (Equations (10)–(15)) as functions of extrusion temperature, with layer height and porosity held at their mid-range values (H = 0.10 mm, ϕ = 60%). As can be seen, the mechanical properties exhibit a maximum at intermediate values, which almost coincides with the minimum values for the geometric properties. This allows us to conclude that, regarding the variation of properties with temperature, there is an equilibrium zone around 215 °C where the best values for both mechanical and geometric properties are obtained simultaneously. However, since mechanical properties also depend on porosity and layer thickness, it is not possible to determine the optimal values for the design and printing factors that simultaneously improve the mechanical and geometric properties of the scaffolds by visual inspection alone. Therefore, it is necessary to use multi-objective optimization methods to achieve this.

#### 3.1.3. Experimental Validation of Regression Models

**Figure 6:**
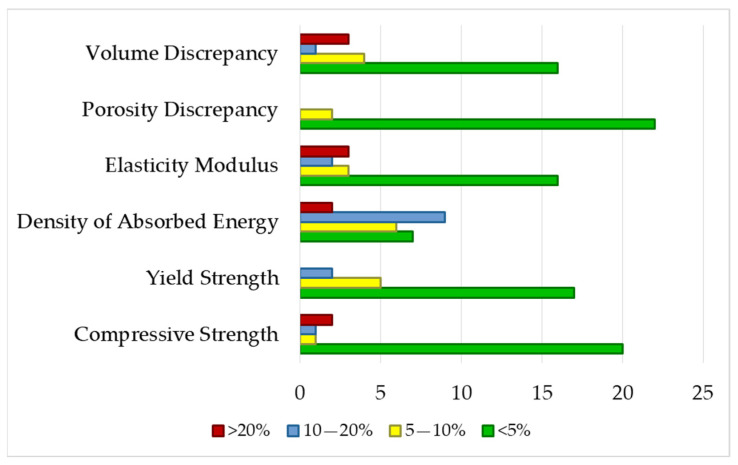
Percentage errors of validation measurements for every property model.

The results from Figure 6 show that in almost all properties, the majority of measurements have relative percentage errors in the range <5%, except for absorbed energy density, where the majority of measurements are concentrated in the 10–20% range. Only a small number of measurements (between two and four, out of a total of 24) have relative percentage errors greater than 20% for each property.

For most properties, the majority of measurements exhibited relative errors below 5%. The exception was absorbed energy density, where most measurements fell within the 10–20% range. Only two–four measurements per property (out of 24 total) showed relative errors exceeding 20%. Overall, these results indicate that approximately 68% of discrepancies are below 5% for all measured properties.

**Figure 7:**
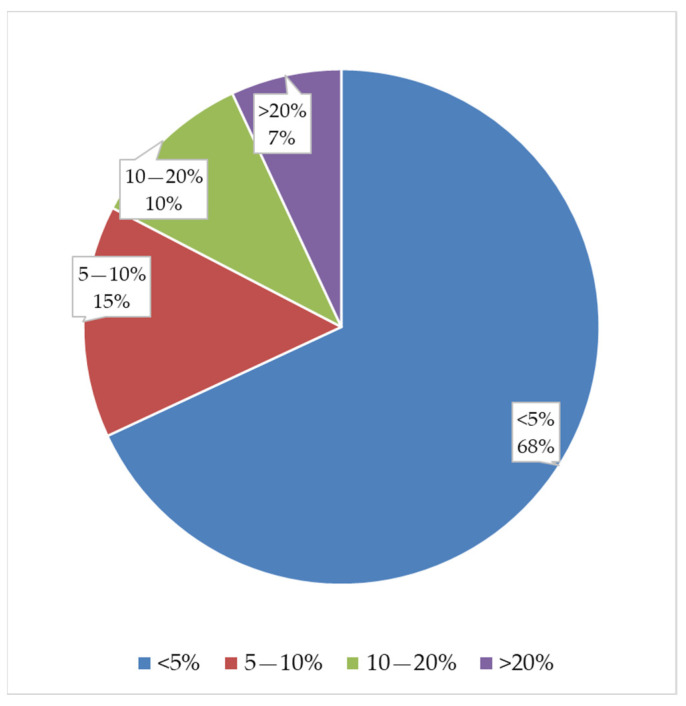
Distribution of relative percentage errors across all validation measurements.

Figure 7 shows the results of the relative percentage errors for the six properties studied. It can be seen that 68% of all measurements have relative percentage errors less than 5%, and only 7% of all measurements have relative percentage errors greater than 20%.

These results confirm that the empirical regression models can estimate mechanical and geometric properties at the extreme points of the experimental factor ranges chosen for validation. Estimates are made with an error of less than 10% in 83% of the measurements and with an error of less than 20% in 93% of the measurements. The experimental validation results allow us to define the applicability limits of the models with greater certainty: layer height: 0.05–0.15 mm; extrusion temperature: 210–220 °C; porosity: 50–70%.

### 3.2. Multi-Objective Optimization Results

#### 3.2.1. Analysis of Correlation Matrices

**Figure 8:**
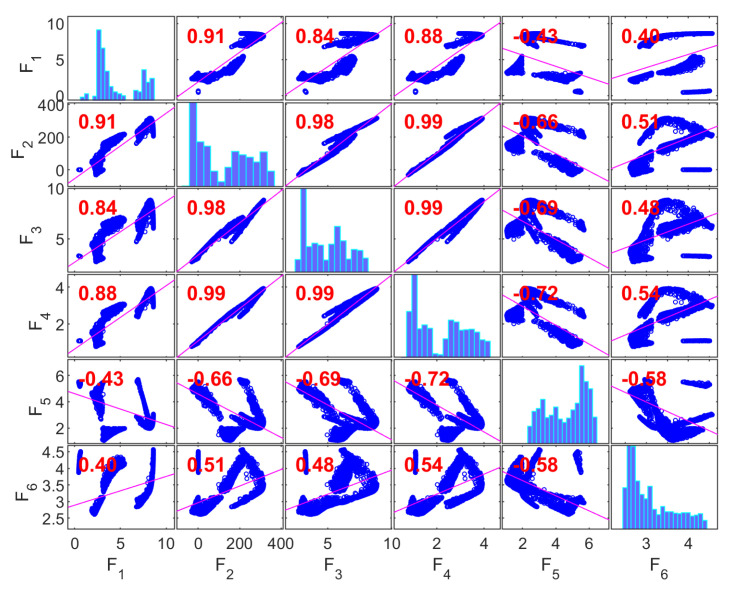
Correlation matrix for the Gyroid structure.

Figure 8 presents the correlation matrix for the Gyroid (G) structure, where the diagonal elements correspond to the marginal distributions of each objective and the off-diagonal elements report pairwise Pearson correlation coefficients between objectives, based on the population generated in the first run of the multi-objective optimization algorithm.

A strong linear association is observed among the mechanical response variables—compressive strength (F1), Young’s modulus (F2), and yield strength (F3)—with correlation coefficients in the range of ~0.91–0.99. This indicates substantial statistical redundancy among these variables within the explored design space. Such coupling is consistent with works showing that the mechanical performance of gyroids is strongly driven by relative density and topology [107,116,117].

The absorbed energy density (F4) also shows very high correlation with F1–F3 (≈0.88–0.99), suggesting that, for the Gyroid topology and the compressive strain range investigated, elastic and post-yield responses vary proportionally. These statistical trends align with observations that gyroids tend to deform through a bending–torsional coupling mechanism, leading to correlated increases in absorbed energy with strength and stiffness [118].

In contrast, the geometric discrepancy variables—porosity discrepancy (F5) and volume discrepancy (F6)—show only moderate correlations with mechanical objectives (≈0.40–0.66). This indicates that, in the parameter ranges studied, variability in manufacturing accuracy is not dominantly linearly associated with mechanical performance, even when gyroids are reported as the most sensitive structure to manufacturing-induced geometrical imperfections that affect the mechanical responses [119].

The moderate correlation between F5 and F6 further indicates that porosity- and volume-related deviations originate from partially independent manufacturing effects. Collectively, these results reveal two statistically distinct objective clusters: a highly correlated mechanical group (F1–F4) and a comparatively independent geometric discrepancy group (F5–F6). This separation supports collapsing the mechanical objectives into a single representative for multi-objective optimization while retaining a geometric fidelity variable.

**Figure 9:**
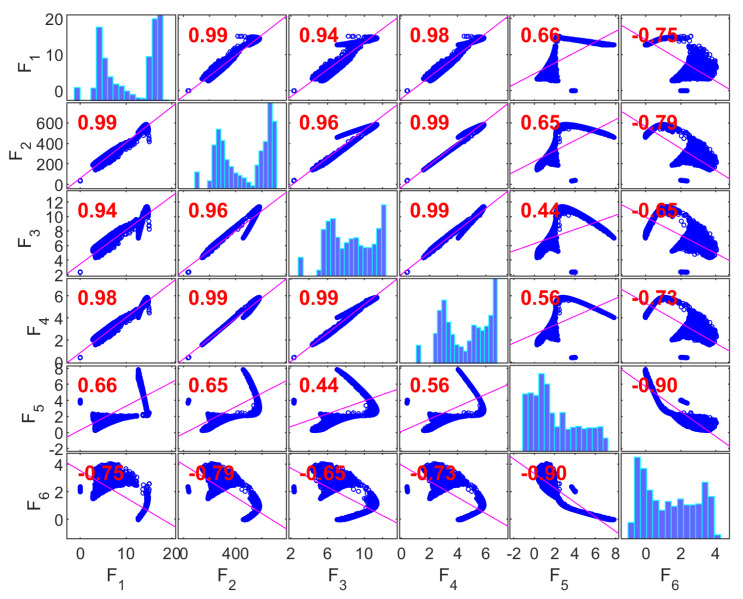
Correlation matrix for the Primitive structure.

Figure 9 shows the correlation matrix for the Primitive (P) structure. As in the Gyroid case, strong correlations are observed among the mechanical objectives; however, the correlation pattern exhibits greater dispersion. The pairwise correlations among compressive strength (F1), Young’s modulus (F2), and yield strength (F3) range from approximately 0.84 to 0.98, indicating substantial but not near-perfect linear dependence.

The absorbed energy density (F4) retains high correlation with other mechanical metrics (≈0.88–0.99), indicating that these variables still co-vary under compression. Nevertheless, these geometries present a stretch-dominated deformation mechanism with a high compression performance but lower energy absorption than gyroids [118]. Another work shows that Primitive structures have better structural continuity, which leads to more uniform load transfer compared to Gyroid structures [120].

The discrepancy variables (F5, F6) again demonstrate weak to moderate correlations with mechanical responses (≈0.40–0.58), confirming that manufacturing deviations exhibit limited linear association with scaffolds’ mechanical performance within the design space. This relative independence has been observed in additive manufacturing contexts, although studies are limited. Geometric discrepancies affect local structural integrity but do not scale predictably with bulk mechanical metrics. The evidence suggests that this relationship is non-linear and depends on the orientation of the structure’s geometric features [121].

Overall, the correlation structure of the Primitive topology reveals two partially independent objective groups, which supports a reduction strategy that selects one representative mechanical metric and one discrepancy metric to capture dominant trade-offs.

**Figure 10:**
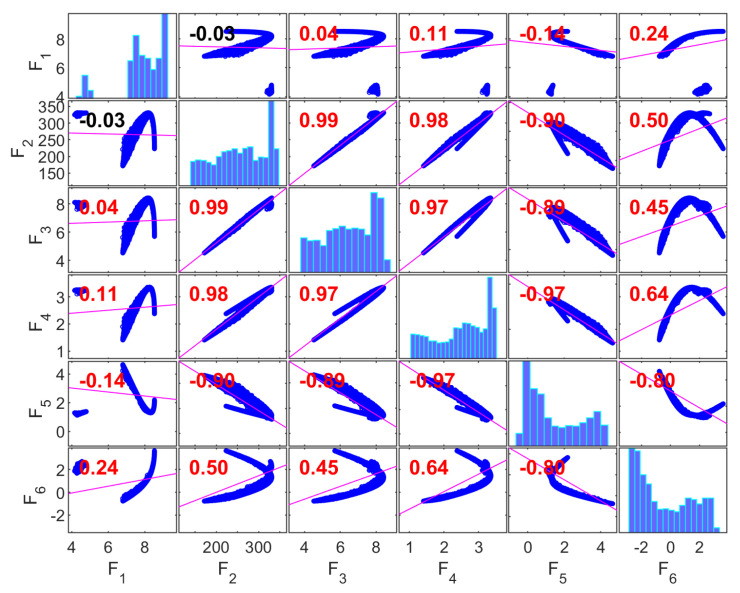
Correlation matrix for the Diamond structure.

Figure 10 presents the correlation matrix for the Diamond (D) structure, showing very high pairwise correlations among compressive strength (F1), Young’s modulus (F2), and yield strength (F3) (≈0.94–0.99), indicating strong coherence among mechanical responses. Recent work supports the claim that Diamond structures consistently outperform the other structures across multiple mechanical properties [121,122].

The absorbed energy density (F4) is also strongly correlated with other mechanical objectives (≈0.90–0.97), suggesting that the elastic and post-yield behaviors scale together in the explored design space. These findings are consistent with studies reporting that in Diamond structures—which exhibit the highest energy absorption efficiency among the three—this correlation is specifically tied to the structure’s ability to sustain high stresses before failure while maintaining structural integrity throughout the deformation process [119].

In contrast, the geometric discrepancy variables (F5 and F6) show weak correlations with mechanical responses (≈−0.14 to 0.44), indicating minimal linear coupling in this dataset. Such decoupling implies that, within the parameter space considered, manufacturing deviations are not the dominant factors governing the linear association patterns among mechanical responses. However, non-linear influences or threshold effects may exist beyond linear correlation measures, as noted in previous works showing that geometric deviations are orientation-dependent [122] and that finite element models can overestimate performance when manufacturing errors are ignored [123]. The modest correlation between F5 and F6 suggests partially independent manufacturing quality effects. Thus, for the Diamond topology, a clear statistical separation exists between a mechanical response cluster (F1–F4) and an independent discrepancy cluster (F5–F6). This is expected, as Diamond structures demonstrate better tolerance to geometric imperfections. Therefore, while correlations exist, the Diamond topology provides some compensation for printing-induced geometric errors [119]. This supports an objective reduction strategy that retains a minimal representative set capturing both mechanical performance and geometric fidelity.

#### 3.2.2. Objective Reduction Results and Interpretation

Table 8 summarizes the results for the application of the objective reduction procedure based on PCA and correlation [85], revealing distinct patterns of essential objectives for each TPMS topology, consistent with the correlation structures previously identified.

**Figure 11:**
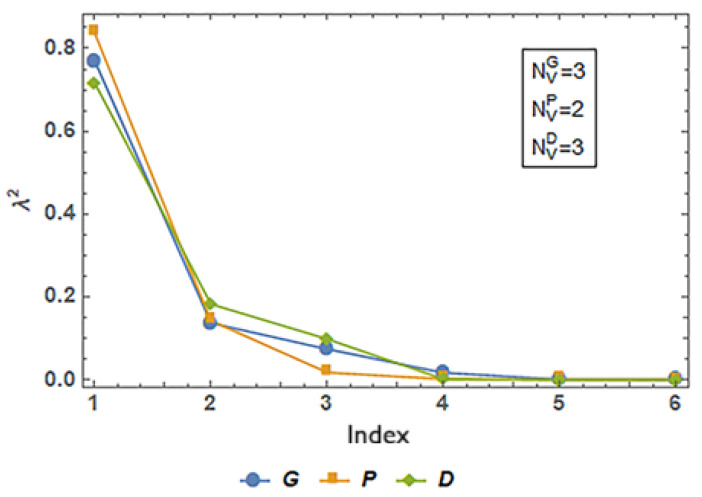
Relative contributions of the main components for each structure.

Figure 11 shows the relative contributions of the principal components to the variance, where the number N_V_ represents the number of principal components that contribute to 95% of the total variance of the correlation, which is the limit set in this work for principal component analysis.

For the Primitive (P) architecture, the algorithm retained F2 (Young’s modulus) and F6 (volume discrepancy) as the minimal essential set. This result reflects the strong collinearity observed among all mechanical responses (F1–F4), indicating that stiffness effectively captures the dominant mechanical axis of variation. Conversely, geometric deviations exhibited weaker association with mechanical properties, with F6 showing sufficient independence to represent dimensional accuracy without redundancy. The essential pair (F2, F6) therefore preserves the primary mechanical and manufacturability characteristics of the P topology.

The Diamond (D) architecture exhibited the most complex objective structure. In the first reduction stage, four objectives—F1 (compressive strength), F2 (Young’s modulus), F4 (energy absorption), and F5 (porosity discrepancy)—were identified as essential, reflecting the weaker mechanical clustering and the partial independence of porosity-related deviations in this topology. A second reduction step further refined the essential set to F2 and F5, indicating that stiffness adequately represents mechanical behavior once initial redundancies are removed, while porosity discrepancy remains the most independent geometric descriptor. This final set captures the primary mechanical and manufacturing dimensions governing the D topology’s multi-objective landscape. As before, the cases of Diamond and Primitive are related, as F2 (Young’s modulus) can be considered the best monotonic descriptor of stiffness-dominated behavior.

The case of the Gyroid structure displayed exceptionally high interdependence among mechanical variables, forming an almost singular mechanical response cluster. Within this group, F4 serves as a comprehensive representative because energy absorption integrates the elastic and plastic contributions relevant to compressive deformation. The F5 objective remained largely decoupled from the mechanical domain, justifying its retention as an independent descriptor of geometric accuracy. The resulting essential set (F4, F5) thus encapsulates both mechanical behavior and dimensional accuracy with minimal redundancy.

#### 3.2.3. Analysis of Pareto Fronts

**Figure 12:**
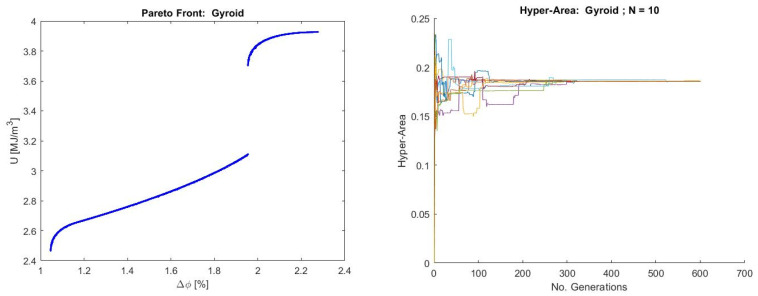
Pareto front for the Gyroid structure (**left**) and hyper-area convergence for 10 runs of NSGA-II (**right**).

The Pareto front for the Gyroid topology (Figure 12) exhibits a well-defined trade-off between energy absorption (F4) and porosity discrepancy (F5), reflecting the interplay between mechanical performance and geometric fidelity inherent to this structure. The front is composed of two smooth, monotonic branches, suggesting the presence of two distinct optimal design regimes, where changes in concavity suggest that the relationship between objectives is not governed by a single principle across the entire front. The lower branch corresponds to solutions with minimal porosity discrepancy and moderate energy absorption, while the upper branch corresponds to higher energy absorption at the expense of increased deviation from the target porosity.

The initial and final portions of the curve reflect severe conflicting objectives, while the middle section represents the more desirable region for decision-makers, as compromises are relatively efficient. The gap in the curve suggests the presence of multimodality in decision space with a forbidden zone where no feasible solution can be attained. The overall shape of the curve implies that the objective functions are non-linear with strong interactions between decision variables. There are essentially two ways to perform decisions: one based on regimes (before and after the jump) and the other based on knee point solutions near the concavity changes where a small degradation in one objective yields a large gain in the other.

The hyper-area convergence plot shows a rapid stabilization within the first 100–200 generations, followed by minor fluctuations and eventual convergence toward a stable value. This fast convergence indicates that the algorithm quickly identifies and approaches the true Pareto front, while the minor fluctuations could suggest the algorithm is fine-tuning around the near optimal front [124]. This also might suggest relatively low multimodality in the objective space, as multimodal problems tend to cause more fluctuating convergence curves. This is also reinforced by the consistency of convergence behavior across repeated runs. However, it must be said that even if the objective space may appear smooth, the decision space could still contain multiple optima [125]. The attainment of a stable hyper-area further confirms that the reduced essential objective set (F4, F5) retains sufficient information to reliably explore and approximate the optimal trade-off space. Overall, the Pareto geometry and hyper-area evolution reinforce the conclusion that the Gyroid exhibits a dominant mechanical response axis and weak coupling to geometric discrepancies.

**Figure 13:**
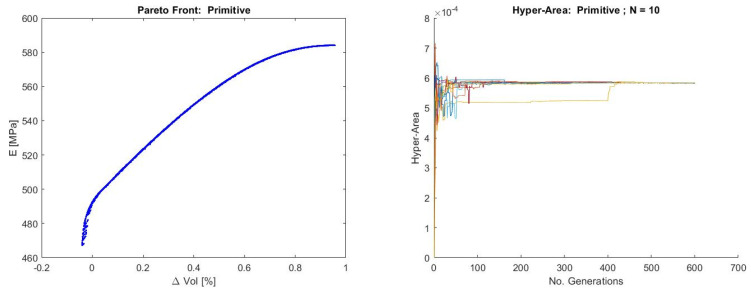
Pareto front for the Primitive structure (**left**) and hyper-area convergence for 10 runs of NSGA-II (**right**).

The Pareto front for the P-structure shown in Figure 13 reveals a highly smooth and strictly monotonic trade-off between Young’s modulus (F2) and volume discrepancy (F6). The continuous, convex shape of the front indicates that increases in stiffness are systematically associated with larger deviations from the target volume, consistent with the strong mechanical coupling and limited geometric–mechanical interaction observed in the correlation analysis. The absence of discontinuities or branching suggests that, in the P topology, geometric fidelity and mechanical stiffness are governed by a single dominant deformation mode, resulting in a particularly well-behaved trade-off landscape, with non-linear but monotonic interactions. All the objective space, for the studied range in decision variables, is feasible.

The hyper-area evolution behaves similarly to the case of the Gyroid structure, with an even faster stabilization and convergence of the algorithm. Regarding these two structures (G and P), it must be noted that while fast, stable convergence suggests efficient front approximation, it cannot definitively characterize multimodality or decision space properties without additional analysis. The hyper-volume’s (hyper-area) complex dependencies make single-metric interpretation insufficient for comprehensive problem understanding [87].

**Figure 14:**
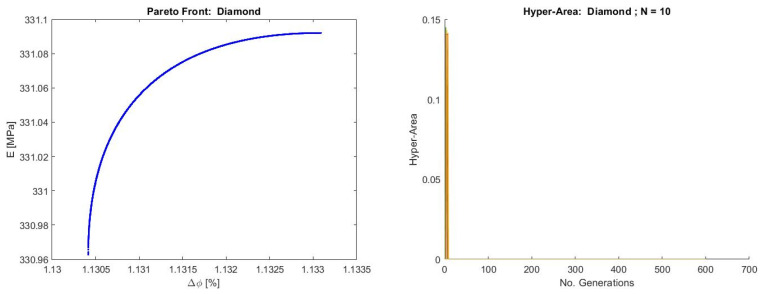
Pareto front for the Diamond structure (**left**) and hyper-area convergence for 10 runs of NSGA-II (**right**).

The Pareto front obtained for the Diamond topology (Figure 14) displays a smooth, connected, and concave well-behaved curve but with contracted decision space. The mapping from decisions to objectives is likely monotonic and involves a single, smooth trade-off curve without local optima regarding Pareto dominance. After objective reduction, the remaining objectives exhibit minimal effective conflict, producing a Pareto set that is mathematically valid but physically shallow: F2 increases only marginally as F5 rises. This reflects the fact that the Diamond structure’s essential behaviors become effectively single-dimensional once redundant objectives are removed.

The hyper-area evolution, at the beginning of the optimization, exhibits a brief non-zero value but rapidly collapses to nearly zero, remaining flat across all subsequent generations. This behavior might indicate critical optimization issues, such as the algorithm’s ability to maintain population diversity or problems with the selection of the reference point for hyper-area calculations. In this case, we think that this behavior stems directly from the small dominated region defined by the reduced objective pair, which is a form of diversity loss. As the front is continuous and smooth, the rapid stabilization could be interpreted as meaning that the optimization landscape is effectively unidimensional and free of multimodality, yielding immediate convergence once the search identifies the narrow feasible region consistent with the Diamond topology’s structural behavior.

Finally, since larger discrepancies, whether in volume or porosity, imply lower print quality and reduced dimensional accuracy, the resulting Pareto fronts present a trade-off when deciding on the properties of a scaffold for a given application. If a higher elastic modulus or higher absorbed energy density is required, a larger discrepancy is obtained. If a scaffold with high dimensional accuracy is needed, then it will have worse mechanical properties. Ultimately, it is up to the decision-maker to decide whether to take a solution at the extremes of the Pareto front or to reach a compromise with intermediate values of dimensional accuracy and mechanical properties.

## 4. Conclusions

### 4.1. General Conclusions

This study has systematically investigated the interplay between design, processing parameters, and the resulting mechanical and geometric properties of PLA scaffolds based on three Triply Periodic Minimal Surface (TPMS) architectures—Gyroid (G), Primitive (P), and Diamond (D)—fabricated via Fused Deposition Modeling (FDM). Through the development of statistically robust quadratic regression models, a detailed analysis of deformation mechanisms, and the application of multi-objective optimization, several overarching conclusions can be drawn.

First, the derived empirical models (Equations (10)–(15)) demonstrate a capability to predict scaffold behavior, with coefficients of determination (R^2^) exceeding 77% and reaching up to 98%. More importantly, these statistically validated models align with established physical principles. The predicted trends—where mechanical properties (compressive strength, Young’s modulus, yield strength, and absorbed energy density) universally decrease with increasing porosity—corroborate classical cellular solid theory. Furthermore, the distinct, topology-dependent deformation mechanisms observed in the stress–strain responses (bending-dominated for G and D, versus stretching/buckling-dominated for P) are accurately reflected in the models’ output, providing both mathematical and physical validation of the regression methodology.

Second, the analysis reveals a fundamental tension, intrinsic to the FDM process of these complex structures: the optimization of mechanical performance often conflicts with the attainment of geometric fidelity. Parameters such as layer thickness and extrusion temperature exhibit non-linear relationships with the mechanical and geometrical properties. An intermediate layer thickness (~0.10 mm) and an extrusion temperature near 215 °C were identified as zones that balance competing demands, but no single parameter set universally maximizes all properties. This inherent conflict necessitates a structured, multi-criteria decision-making approach, as visual inspection or single-objective tuning is insufficient.

Third, a pivotal finding of this work is the successful application of objective reduction techniques, which clarified the core trade-off landscape for each TPMS type. Correlation analysis powered by principal component analysis revealed that the six original objectives collapse into distinct, minimal essential sets:

- For the Gyroid (G) structure, the trade-off is captured between Absorbed Energy Density (F4) and Porosity Discrepancy (F5).
- For the Primitive (P) structure, the essential conflict lies between Young’s Modulus (F2) and Volume Discrepancy (F6).
- For the Diamond (D) structure, the reduced set converges to Young’s Modulus (F2) and Porosity Discrepancy (F5).

This reduction underscores that while mechanical responses (strength, stiffness, energy absorption) are highly correlated within each topology, creating a dominant “mechanical performance axis,” the geometric imperfections (porosity and volume discrepancies) represent a largely independent “manufacturability axis.” The specific pairing (e.g., F4 for G, F2 for P and D) is topology-specific, reflecting the underlying deformation mechanics: energy absorption is paramount for the tough, bending Gyroid, while stiffness is representative for the Primitive and Diamond.

Finally, the generated Pareto fronts provide a powerful decision-support tool for designers. Each front—smooth and convergent for P and G, and highly compact for D—graphically defines the achievable limits of performance. The shape of the Gyroid’s front, for instance, suggests two distinct design regimes, while the Primitive’s front shows a continuous, monotonic trade-off. The key practical implication is that selecting a scaffold for a specific biomedical or engineering application is not a matter of finding a universally “best” configuration but of navigating these topology-specific trade-offs. A designer prioritizing high energy absorption for impact protection might select a Gyroid solution from the upper branch of its Pareto front, accepting higher porosity discrepancy. Conversely, an application requiring precise dimensional tolerances and high stiffness might lead to a Diamond or Primitive solution from the lower-discrepancy region of their respective fronts.

In summary, this research provides a validated, quantitative framework linking FDM process parameters and design variables to functional outcomes for TPMS scaffolds. It demonstrates that through statistical modeling and multi-objective optimization, the complex, non-linear relationships governing these structures can be translated into clear, actionable design guidelines. The findings emphasize that optimal scaffold design is inherently a compromise, guided by the specific mechanical requirements and allowable manufacturing tolerances of the intended application. Future work can build upon this framework by incorporating additional materials, dynamic loading conditions, and in vivo biological performance criteria.

### 4.2. Directions for Future Work

While this study establishes a robust quantitative framework for the design of TPMS scaffolds, it naturally opens several lines for further investigation. A direct extension would involve the experimental validation of the Pareto-optimal configurations identified for each topology. Manufacturing and mechanically testing these predicted optimum scaffolds would serve as the definitive proof of the optimization framework’s practical utility and could reveal subtle interactions between parameters not fully captured by the regression models. Furthermore, this research focused on quasi-static compressive loading. Future work should characterize scaffold performance under dynamic or cyclic loading conditions, which are more representative of in vivo physiological environments for bone tissue engineering. Assessing fatigue life, damping characteristics, and long-term structural integrity would provide a more comprehensive understanding of functional suitability.

Beyond the limitations in applicability of the obtained mathematical models and optimization results, dictated by the experimental conditions and the selected range of decision variables, the methodology presented in this work could be extended to include the effects of different materials, 3D printers, and printing processes, as well as other important variables.

Beyond mechanical characterization, the logical progression of this work lies in integrating biological evaluation criteria into the multi-objective optimization schemes. The current objectives of mechanical performance and geometric fidelity could be augmented—or partially substituted—by metrics of biological functionality. These might include in vitro assessments of cell adhesion, proliferation, and differentiation on scaffolds with varying topology and surface morphology, or computational fluid dynamics (CFD) analyses of permeability and shear stress for nutrient transport. Such a multidisciplinary approach would shift the optimization goal from creating a structurally efficient scaffold to designing a truly bioactive construct, where the trade-offs between mechanical support, manufacturable accuracy, and biomimetic stimulation are properly balanced. This would represent a significant step towards the clinically informed, application-driven design of advanced biomedical implants.

## Figures and Tables

**Table 1:** C-level sets for TPMS porosities.

TPMS	Porosity [%]	C-Level Set
P	50	0
P	60	−0.347
P	70	−0.694
G	50	0
G	60	−0.306
G	70	−0.608
D	50	0
D	60	−0.167
D	70	−0.335

**Table 2:** Default printing parameters.

Parameter	Default Value
Wall thickness [mm]	0.8
Print speed [mm/s]	20
Platform temperature [°C]	60
Adhesion platform	Raft
Nozzle diameter [mm]	0.2
Retraction speed [mm/s]	40
Retraction distance [mm]	7
Infill speed [mm/s]	15

**Table 3:** Consulted reviews for experimental design.

Reference	Year	Total	Useful	Scope
[55]	2022	80	80	2010–2022
[57]	2021	22	22	2006–2021
[58]	2019	23	23	2005–2019
[43]	2019	13	13	2001–2019
[59]	2019	250	100	2005–2019
[60]	2018	293	49	2008–2017
[61]	2015	22	22	2003–2015

**Table 4:** Factor levels for regression models.

Factor	Type	Low	Medium	High
Extrusion temperature (*T*) [°C]	Continuous	210	215	220
Layer thickness (*H*) [mm]	Continuous	0.05	0.10	0.15
Porosity (*ϕ*) [%]	Continuous	50	60	70
Type of TPMS [*S*]	Nominal	P	G	
Structure P		1	0	
Structure G		0	1	
Structure D		0	0	

**Table 5:** Formulation of the optimization problem.

Objective Functions	Objective	Limits (Restrictions)
$F_{1}:\sigma_{C}\left(T,H,\phi ,S\right)$	Maximize	210≤T≤220 0.05≤H≤0.15 50≤ϕ≤70 $S=\left\{G,P,D\right\}$
$F_{2}:E\left(T,H,\phi ,S\right)$	Maximize	
$F_{3}:\sigma_{Y}\left(T,H,\phi ,S\right)$	Maximize	
$F_{4}:U\left(T,H,\phi ,S\right)$	Maximize	
$F_{5}:∆\phi \left(T,H,\phi ,S\right)$	Minimize	
$F_{6}:∆Vol\left(T,H,\phi ,S\right)$	Minimize	

**Table 6:** Parameter values for NSGA-II.

**Modified Parameters**	**Value**
FunctionTolerance	0
ConstraintTolerance	0
PopulationSize	1000
MaxStallGenerations	500
**Default Parameters**	**Value**
FitnessScalingFcn	Rank
SelectionFcn	Tournament
EliteCount	ceil(0.05 × PopulationSize)
MutationFcn	Adaptive Feasible
CrossoverFcn	Intermediate

**Table 7:** Summary of statistical parameters for regression models. DL and DU are the lower and upper critical values for the Durbin–Watson test, respectively; NT is the number of terms.

Model	$R^{2}$	$R_{adjusted}^{2}$	Durbin–Watson			
	Sig.	Sig.	NT	D_L_	D_U_	D_W_
$\sigma_{C}$	98.00	97.82	12	1.54677	1.89739	2.14296
E	79.64	78.52	11	1.56315	1.87994	1.96105
$\sigma_{Y}$	78.93	77.24	9	1.59547	1.84573	1.91706
U	91.37	90.82	9	1.59547	1.84573	1.97504
∆Vol	91.12	90.00	16	1.48007	1.96947	2.16126
∆Por	81.59	79.44	15	1.49692	1.95112	1.76237

**Table 8:** Results for the objective reduction procedure.

TPMS	Essential Obj. (1st Run)	Essential Obj. (2nd Run)	F1: Compressive strength F2: Young’s modulus F3: Yield strength F4: Absorbed energy density F5: Porosity discrepancy F6: Volume discrepancy
G	F4 and F5	-	
P	F2 and F6	-	
D	F1, F2, F4, and F5	F2 and F5	

## Data Availability

The original contributions presented in this study are included in the article. Further inquiries can be directed to the corresponding author.

## Abbreviations

The following abbreviations are used in this manuscript:

PLA	Poly (lactic acid)
FDM	Fused Deposition Modeling
RSM	Response Surface Methodology
ANOVA	Analysis of Variance
BBD	Box–Behnken Design
CCD	Central Composite Design
FDA	Food & Drug Administration
AM	Additive Manufacturing
GRA	Gray Relational Analysis
ANN	Artificial Neural Network
ANFIS	Adaptive Neuro-Fuzzy Inference System
NSGA	Non-dominated Sorting Genetic Algorithm
TPMS	Triply Periodic Minimal Surface
VIF	Variance Inflation Factor
MOO	Multi-Objective Optimization
PSO	Particle Swarm Optimization
DOE	Design of Experiments
CAD	Computed Aided Design
